# Enhanced Exciton Effect and Singlet Oxygen Generation Triggered by Tunable Oxygen Vacancies on Bi_2_MoO_6_ for Efficient Photocatalytic Degradation of Sodium Pentachlorophenol

**DOI:** 10.3390/ijms232315221

**Published:** 2022-12-02

**Authors:** Xiao Xu, Xianglong Yang, Yunlong Tao, Wen Zhu, Xing Ding, Junjiang Zhu, Hao Chen

**Affiliations:** 1 Hubei Key Laboratory of Biomass Fibers and Eco-Dyeing & Finishing, College of Chemistry and Chemical Engineering, Wuhan Textile University, Wuhan 430200, China; 2 College of Science, Huazhong Agricultural University, Wuhan 430070, China; 3 National Reference Laboratory for Agricultural Testing (Biotoxin), Laboratory of Quality and Safety Risk Assessment for Oilseed Products (Wuhan), Key Laboratory of Detection for Mycotoxins, Quality Inspection and Test Center for Oilseed Products, Ministry of Agriculture and Rural Affairs, Oil Crops Research Institute, Chinese Academy of Agricultural Sciences, Wuhan 430062, China

**Keywords:** oxygen vacancy, exciton effect, Bi_2_MoO_6_, sodium pentachlorophenate, photocatalysis

## Abstract

Construction of the tunable oxygen vacancies (OVs) is widely utilized to accelerate molecular oxygen activation for boosting photocatalytic performance. Herein, the in-situ introduction of OVs on Bi_2_MoO_6_ was accomplished using a calcination treatment in an H_2_/Ar atmosphere. The introduced OVs can not only facilitate carrier separation, but also strengthen the exciton effect, which accelerates singlet oxygen generation through the energy transfer process. Superior carrier separation and abundant singlet oxygen played a crucial role in favoring photocatalytic NaPCP degradation. The optimal BMO-001-300 sample exhibited the fastest NaPCP degradation rate of 0.033 min^−1^, about 3.8 times higher than that of the pristine Bi_2_MoO_6_. NaPCP was effectively degraded and mineralized mainly through dechlorination, dehydroxylation and benzene ring opening. The present work will shed light on the construction and roles of OVs in semiconductor-based photocatalysis and provide a novel insight into ROS-mediated photocatalytic degradation.

## 1. Introduction

The excessive discharge of pesticide pollutants produced in agricultural activity has brought serious environmental pollution and caused a heavy burden on the ecological system [1,2,3]. Among all pesticide pollutants, sodium pentachlorophenate (NaPCP) has been listed as a priority contaminant by the U.S. Environmental Protection Agency due to its bio-refractory and high biological toxicity [4]. It is of great urgency and significance to develop novel technology for efficient NaPCP removal. Compared with other advanced oxidation processes for NaPCP degradation, photocatalytic technology is regarded as one of the most potentially effective methods of pollution control and environmental remediation. It works by utilizing light-induced reactive oxygen species (ROS) to degrade organic contaminants [5,6,7,8].

Until now, numerous semiconductors including metal oxides (TiO_2_, BiOBr and Bi_2_MoO_6,_ etc.) [9,10,11] and organic polymers (C_3_N_4_, MOF and COF, etc.) [12,13,14] have been chosen as the candidates for NaPCP degradation. However, photocatalytic NaPCP degradation still suffers from a lack of active species and unsatisfying reaction efficiency [15,16]. To improve the reactivity for NaPCP degradation, multifarious modification strategies have been explored in recent years to strengthen the photocatalytic activity of semiconductors, such as heterojunction construction, co-catalyst modification and defect engineering [17,18,19,20].

Oxygen vacancies (OVs), the typical anion defects, have been regarded as an effective modification to enhance the photocatalytic reactivity [21,22]. The introduced OVs are capable of extending the light absorption region of semiconductors and accelerating the carrier migration and separation of charge carriers [23,24]. Wei et al. employed transient absorption spectroscopy to confirm that OVs on the WO_3_ surface were enabled to induce electron trapping states and inhibit the direct recombination of photogenerated carriers [25]. More importantly, OV-mediated electrons in localized states led to more photogenerated electrons gathering, serving as the active sites for molecular oxygen and reactive substrate activation [26]. Li et al. systematically explored the impacts of OVs on the optical absorption of BiOCl and molecular oxygen activation [27]. The results indicated that the OVs on the catalyst surface can selectively activate oxygen to •O_2_^−^, which dominated the dechlorination process of NaPCP, thus achieving the excellent photocatalytic NaPCP degradation [27]. Their subsequent research found that different facet-dependent OVs exhibited different water adsorption configurations in the form of chemical adsorption or dissociation adsorption, which determined the ability of adsorbed water molecules to be oxidized by holes [28].

Considerable research efforts have been devoted to investigating the role of OVs in ROS generation from two aspects, including the electron transfer mechanism induced by charge-carrier behavior and the energy transfer mechanism caused by the exciton effect [29,30,31,32,33]. The electron transfer process results from the separated carriers’ transfer to molecular oxygen, thus realizing the activation of molecular oxygen to generate ROS (such as •O_2_^−^, H_2_O_2_ and •OH). The energy transfer process originates from the transformation from a high-energy singlet exciton to the triple excited state via the inter-system crossover, accompanying molecular oxygen activation to produce ^1^O_2_ [34]. As reported, the OV-mediated electron capture state on the semiconductor surface can activate O_2_ to produce • O_2_^−^ and H_2_O_2_ through the electron transfer process utilizing localized electrons [35]. The introduced OVs also increase the highly delocalized valence electrons at adjacent atoms, which will further weaken the charge shielding effect and enhance the electron-hole interaction, thus endowing the catalyst with increased exciton binding energy [36]. Achieving tunable OVs is of great significance for arranging the exciton or carrier behavior. Although many modification strategies have been developed to introduce tunable OVs, including solvothermal processes and reduction with UV illumination and doping, these are complicated and uncontrollable. There is therefore an urgent need to solve the significant challenge of achieving the accurate and controllable construction of OVs.

Herein, we choose Bi_2_MoO_6_, a typical layered Aurivillius material, as the photocatalyst model, and realize the mild and controllable construction of OVs on the Bi_2_MoO_6_ surface via adjusting the calcination temperature under H_2_/Ar atmosphere. The tunable OVs introduced here are proven to have several function schemes, not only endowing Bi_2_MoO_6_ with efficient carrier separation and an optimized band gap via the constructing defects level, but also enabling the weak charge screening effect, which is of great significance for the enhanced exciton effect and ^1^O_2_ production through the energy transfer process. Subsequently, the photocatalytic performance of the NaPCP degradation is evaluated to explore the contribution of different active species in the degradation process. Finally, a possible degradation mechanism of NaPCP is proposed with the main degradation step of dechlorination, dehydroxylation and benzene ring oxidation and breakage based on the in-situ DRIFTS experiments.

## 2. Results and Discussion

### 2.1. Characterization of the Samples

**Figure 1:**
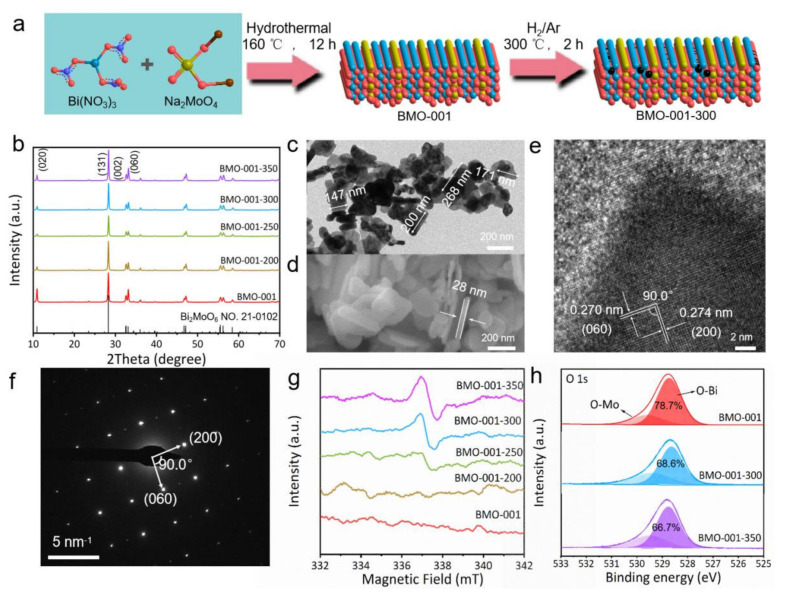
(**a**) Schematic illustration of BMO-001-300 preparation with calcination treatment; (**b**) XRD patterns of as-prepared Bi_2_MoO_6_ samples; TEM (**c**), SEM (**d**), HRTEM (**e**) and SAED (**f**) images of BMO-001-300; (**g**) EPR spectra of oxygen vacancies on as-prepared Bi_2_MoO_6_ samples; (**h**) high-resolution XPS spectra of O 1s for BMO-001, BMO-001-300, BMO-001-350.

As shown in Figure 1a, Bi_2_MoO_6_ with (001) facet exposed was firstly prepared by adjusting the pH of the reaction solution referring to our previous work [37]. Subsequently, OVs on BMO-001 were in-situ constructed by calcination treatment at 300 °C under H_2_/Ar atmosphere to attain BMO-001-300. The as-prepared samples were characterized by XRD to investigate the crystal phase and structure in Figure 1b. All samples exhibited clear diffraction peaks, which were consistent with the characteristic diffraction peaks of tetragonal phase Bi_2_MoO_6_ (PDF No. 21-0102). This result indicated that pure Bi_2_MoO_6_ photocatalysts were successfully prepared via the typical hydrothermal method, and the subsequent calcination treatment hardly caused any change in the crystal structure of the Bi_2_MoO_6_ sample. In addition, the high intensity of the (002) diffraction peak implied the preferential growth and exposure of the (001) facet on Bi_2_MoO_6_, which was consistent with the result reported in our previous work [16,37]. The micro morphologies of all samples were depicted in Figure S1 with TEM images. The specific surface area of these samples also had no obvious change (Figure S2). The as-prepared samples exhibited similar nanosheet morphology, demonstrating that the calcination treatment process did not cause any destruction to the structure and size of Bi_2_MoO_6_ nanosheets. As shown in Figure 1c,d, the BMO-001-300 nanosheets exhibited a width of 200 nm and a thickness of 28 nm. HRTEM images of BMO-001-300 displayed distinct orthorhombic lattice fringes with spaces of 0.270 nm and 0.274 nm, corresponding to the interplanar distance of (060) planes and (200) planes, thereby suggesting BMO-001-300 is indeed exposed with the (001) facets (Figure 1e). Identical conclusions were obtained in the selected area electron diffraction (SAED) patterns of BMO-001-300 (Figure 1f).

To confirm the existence of OVs on BMO-001-300, the EPR test was performed by monitoring the single–electron-induced paramagnetic signal of the as-prepared samples. As observed in Figure 1g, a continuously enhanced paramagnetic signal at g = 2.003, corresponding to the OVs signal, gradually appeared with the increase of the calcination temperature during the synthesis process. This result conveyed the fact that the strategy for in-situ construction of OVs in this work was practicable. The XPS test was conducted to analyze the surface electronic states and chemical compositions of samples (Figure S3 and Figure 1h). The high-resolution XPS spectra of Bi 4f and Mo 3d over BMO-001 indicated that no change occurred on BMO-001-300 and BMO-001-350 (Figure S3b,c). The high-resolution XPS spectra of O 1s were divided into two peaks corresponding to the Bi-O (528.7 eV) and Mo-O bands (529.6 eV) (Figure 1h). Notably, the peak area of the Bi-O signals over BMO-001-300 and BMO-001-350 was much weaker than that of BMO-001, thereby implying that the OVs of BMO-001-300 and BMO-001-350 resulted from the escape of O atoms connected with Bi atoms.

### 2.2. Photocatalytic Activity

**Figure 2:**
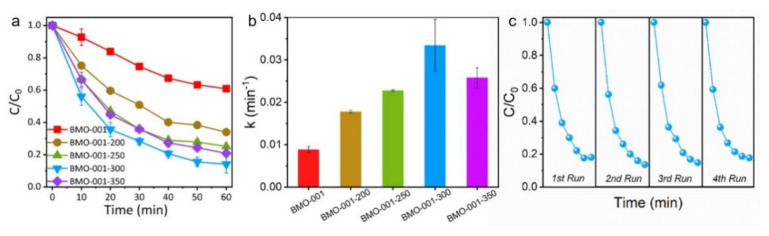
Photocatalytic NaPCP degradation performance curve (**a**) and reaction rate (**b**) on as-prepared Bi_2_MoO_6_ samples under visible light irradiation; (**c**) recycle performance of BMO-001-300 for photocatalytic NaPCP degradation.

As reported numerous times in the literature, OVs played a vital role in improving photocatalytic performances by inducing coordination unsaturation and electron localization [16,19,30,38]. Thus, photocatalytic degradation of NaPCP with visible light irradiation was conducted to evaluate the roles of surface OVs. As exhibited in Figure 2a, all catalysts treated by calcination performed superior photocatalytic activities for NaPCP degradation, implying that the OVs induced by the calcination treatment used on BMO-001-X (X = 200, 250, 300, 350) could be a great impetus for boosting the photocatalytic performance. Compared with the reported catalysts, the photocatalytic performance of the optimized BMO-001-300 also showed a significant advantage (Table S1). According to the first-order translation kinetics, the rate constant of the reaction was fitted and calculated. Briefly, the reaction kinetic constant of NaPCP degradation on BMO-001-300 was 0.033 min^−1^, which is slightly higher than BMO-001-350 and 3.8 times that of the pure BMO-001 (0.009 min^−1^) (Figure 2b). The above results suggest that surface OVs could accelerate the photocatalytic process, but that excessive OVs restrain the degradation process, possibly because excessive OVs act as recombination traps of electron-hole pairs. As shown in Figure 2c, the photocatalytic activity for NaPCP degradation hardly decayed after four recycling runs. Furthermore, no obvious changes were observed in the XRD patterns and the XPS spectra of BMO-001-300 after four runs of photocatalytic tests (Figure S4), indicating that BMO-001-300 kept excellent recycle stability.

### 2.3. Mechanisms of Photocatalytic NaPCP Degradation on BMO-001-300

**Figure 3:**
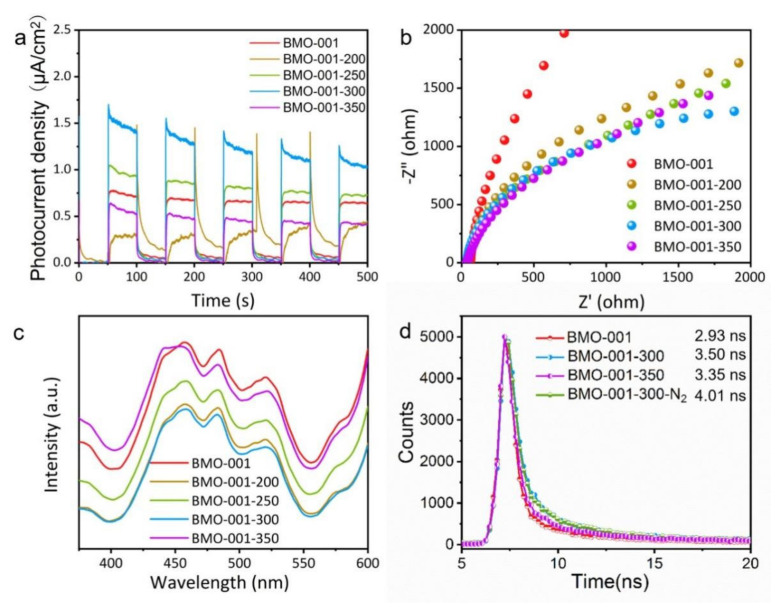
Photocurrent tests (**a**), EIS (**b**) and steady-state photoluminescence spectra (**c**) and time-resolved transient photoluminescence decay spectra (**d**) of as-prepared Bi_2_MoO_6_ samples.

As shown in Figure S5, these photocatalysts exhibited gradually enhanced light absorption with the OVs increasing, demonstrating that the introduced OVs expanded the light absorption range. As exhibited in the photocurrent response test, the OV-modified BMO-001-300 sample displayed higher photocurrent intensity compared with its counterparts, indicating that the OVs favored the internal charge separation efficiency (Figure 3a). Besides, BMO-001-300 presented a smaller radius of EIS Nyquist plots than BMO-001, suggesting a lower charge transfer resistance and a greater impetus for electron transfer (Figure 3b). It contributed to the accelerated charge transport promoted by the OVs. In addition, the PL behavior was monitored to acquire the related information for charge separation (Figure 3c). The lower PL emission intensity on BMO-001-300 meant a lower recombination probability of the photogenerated carriers, confirming that the existence of OVs hindered the recombination of photogenerated e^−^/h^+^ pairs. To reveal the OV-mediated electron transfer process, TR-PL was performed to test the carrier lifetime. As shown in Figure 3d, the electron lifetime of the samples followed the order of BMO-001-300 > BMO-001-350 > BMO-001. The result indicated that the appropriate OVs could favor effective electron separation, greatly extending the electron lifetime, while excessive OVs accelerated carrier recombination. Interestingly, when oxygen in the environment was isolated, the electron lifetime was significantly prolonged, implying that photoelectron transfer was conducted with oxygen in the form of electron transfer or exciton recombination in the photocatalytic process.

**Figure 4:**
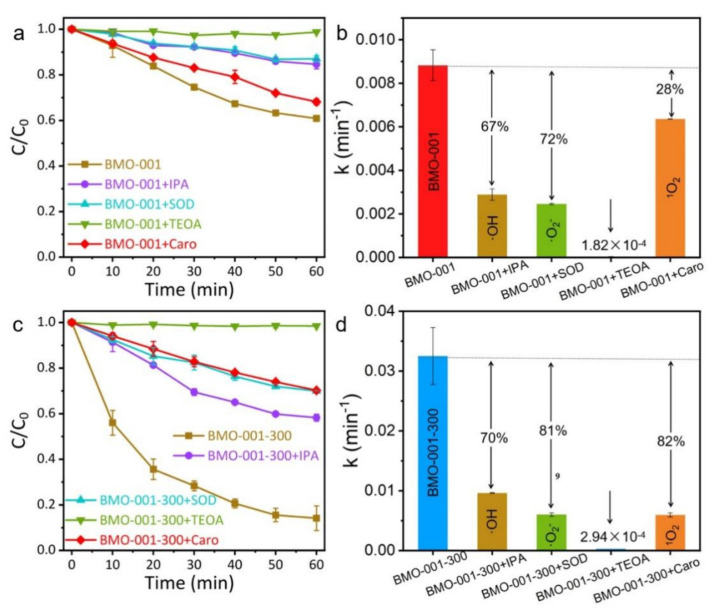
Photocatalytic NaPCP degradation performance curve and reaction rate on BMO-001 (**a**,**b**) and BMO-001-300 (**c**,**d**) with different scavengers (TEOA→h^+^, IPA→•OH, SOD→•O_2_^−^, caro→^1^O_2_) under visible light irradiation, respectively.

Photocatalytic oxygen or water molecule activation produced abundant active species with excellent reactivity, displaying a great impetus for photocatalytic reaction. To explore the dominant roles of these active species in the reaction, the photocatalytic NaPCP degradation of BMO-001 and BMO-001-300 were evaluated with isopropanol (IPA), superoxide dismutase (SOD), natural β-carotene (Caro), and triethanolamine (TEOA) as the •OH, •O_2_^–^, ^1^O_2_ and holes scavengers, respectively (Figure 4). As for both BMO-001 and BMO-001-300 photocatalysts, the degradation rate of NaPCP significantly decreased with TEOA capturing the holes, suggesting that the holes became the most important active species by activating NaPCP and lowering the reaction barrier. As reported in several works [1,16,39], •OH and •O_2_^–^ also played an indispensable role in the degradation process. The contribution rates of •OH and •O_2_^–^ in the photocatalytic system of BMO-001-300 reached 70% and 81%, respectively, which demonstrated few differences with that of BMO-001, suggesting that the OVs did not possess a positive tendency in the degradation process triggered by •OH and •O_2_^–^. It is noteworthy that the absence of ^1^O_2_ inhibited 82% of NaPCP degradation for BMO-001-300, which was much higher than the 28% of BMO-001, revealing that ^1^O_2_ was the critical factor to improve photocatalytic reactivity.

**Figure 5:**
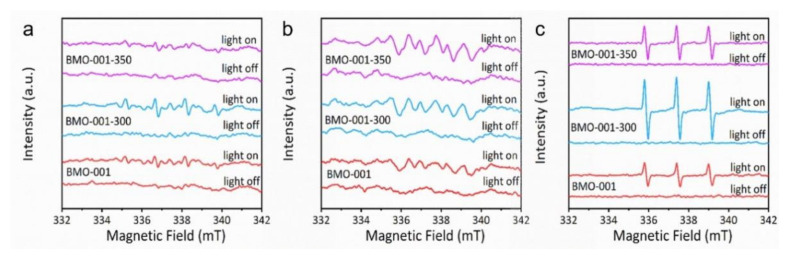
EPR signal of DMPO-•OH (**a**), DMPO-•O_2_^−^ (**b**) and TEMP-^1^O_2_ (**c**) for BMO-001, BMO-001-300 and BMO-001-350 under visible light irradiation.

Subsequently, we utilized EPR spin-trapping tests to detect the photogenerated ROS, including •OH, •O_2_^–^ and ^1^O_2._ As shown in Figure 5a,b, slight enhancement was observed in the quadruple peak with the intensity ratio of 1:2:2:1 and the six-fold peak corresponding to the addition products of DMPO-•OH and DMPO-•O_2_^–^ over BMO-001-300, which proved that OVs played an insignificant role in accelerating •OH and •O_2_^–^ production by activating oxygen or water molecules through the electron transfer process. Interestingly, when TEMP was added to capture ^1^O_2_, a clear triple peak signal with the intensity ratio of 1:1:1 was generated. This TEMP-^1^O_2_ signal of BMO-001-300 was much stronger than that of BMO-001 and BMO-001-350 (Figure 5c). The above results indicated that an appropriate amount of OVs can promote the production of ^1^O_2_, which may be attributed to the fact that the OV-modified BMO-001-300 material was prone to induce excitonic enhancement. This EPR test result also supported the scavenger experiment for NaPCP degradation, confirming the crucial role of multiple ROS, especially ^1^O_2_, in the photocatalytic degradation process.

**Figure 6:**
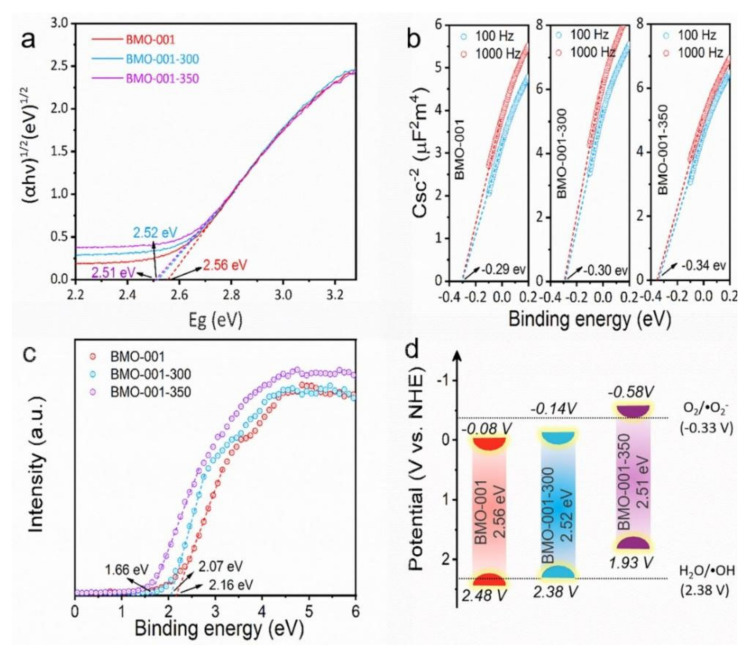
The plots of (αhν)^1/2^ versus photon energy (hν) (**a**), Mott–Schottky plots (**b**), VB-XPS (**c**) and a schematic illustration of the band structure (**d**) of BMO-001, BMO-001-300 and BMO-001-350.

To verify the feasibility of the OVs on the Bi_2_MoO_6_ surface to activate oxygen and water molecules to generate ROS, we tested and calculated the band positions of the samples through a series of characterizations. Based on the diffuse reflectance ultraviolet-visible spectra, the plots (αhv)^1/2^ versus the energy of absorbed light were estimated to calculate the bandgap energies of the samples (Figure 6a). The sample with more oxygen vacancies obtained a narrower band gap, which should be ascribed to the OV-induced defect levels [18]. The Mott–Schottky test was employed to determine the flat band potentials of samples (Figure 6b). The flat band potentials of BMO-001, BMO-001-300 and BMO-001-350 were −0.29, −0.30 and −0.34 V (vs. Ag/AgCl), corresponding to 0.32, 0.31 and 0.27 V (vs.NHE), which were nearly equal to their Fermi level for n-type semiconductors. Figure 6c illustrated the VB-XPS spectra of all samples, indicating that the energy gaps between their Fermi level (E_f_) and VB were 1.66, 2.07 and 2.16 eV for BMO-001, BMO-001-300 and BMO-001-350, respectively. The above results enabled us to pinpoint the VB position of the sample directly and acquire the CB position combined with the band gap values (Table S2). For BMO-001, its CB potential was lower than -0.33 V vs. NHE for the conversion from O_2_ to •O_2_^–^, which was not negative enough to activate oxygen. Once OVs were introduced, the obtained BMO-001-300 and BMO-001-350 not only possessed more activation sites, but also exhibited more negative CB positions, which facilitated the transfer of photogenerated electrons to oxygen and may activate molecular oxygen (Figure 6d). Considering the unique electron trap effect of OVs, the OV concentration has a significant influence on the regulation of exciton and carrier behavior [36]. When appropriate OVs were introduced using a calcination treatment at 300 °C, BMO-001-300 maintained a notable Coulomb-interaction-mediated excitonic effect, which resulted from the attenuated dielectric screening induced by the OVs. Excess OVs provided more electron localization centers, enabled the acceleration of the electron transfer behavior of carrier separation, and correspondingly weakened the energy transfer process induced by the excitons, finally resulting in the production of increased •O_2_^–^ and decreased ^1^O_2_.

**Figure 7:**
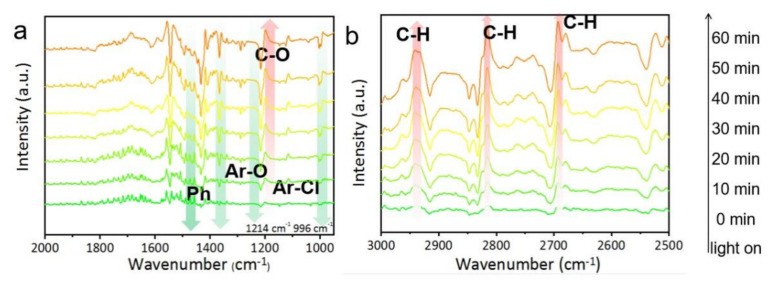
In situ DRIFTS spectra of photocatalytic NaPCP degradation in the range of 2000–1000 cm^−1^ (**a**) and 3000–2500 cm^−1^ (**b**) with BMO-001-300 under irradiation.

In-situ DRIFTS experiments were conducted to dynamically detect the reaction intermediates and track the possible photodegradation route. In general, the negative bands and positive bands in in-situ DRIFTS spectra represented the depletion of reactants and the accumulation of products during the reaction, respectively. Both negative and positive peaks in the DRIFTS spectra of the two systems were observed in Figure 7. These increased negative bands at 1450–1600 cm^−1^ were attributed to the vibration peaks of the consumed benzene ring skeleton, which suggested that the carbon skeleton of NaPCP was continuously attacked and decomposed under visible light irradiation. Besides, the negative peaks at 996 cm^−1^ and 1214 cm^−1^ were observed in Figure 7a, corresponding to the stretching vibration of the aromatic C-Cl bond and C-O bond within NaPCP molecules, respectively. These results imply that the C-Cl bond and C-O bond of adsorbed NaPCP were consumed over BMO-001-300 under illumination. As described in Figure 7b, it was obvious that there were three positive bands located in the ranges of 2760–2950 cm^−1^ and the absorption intensity increased over time, which corresponded to the vibrations of the boosted C-H bond.

Based on the above in-situ DRIFTS analyses, the mechanism of photocatalytic NaPCP degradation over OV-modified BMO-001-300 is summarized. Considering the great contribution of abundant active species, especially holes and ^1^O_2_, the adsorbed NaPCP is easier to degrade and mineralize. NaPCP was decomposed mainly through the following three paths, including dehydrogenation, dechlorination and benzene ring decomposition. Meanwhile, the significantly enhanced C-H peaks of the in-situ DRIFTS spectrum indicated that the chemically stable NaPCP was gradually degraded and transformed into non-toxic organic small molecules, and finally completely mineralized.

### 2.4. Conclusions

In summary, tunable OV construction on the Bi_2_MoO_6_ surface was completed through the modulation of the temperature in the calcination treatment. The introduced OVs can not only promote carrier separation and optimize the band gap structure, but also greatly drive the exciton effect, thus generating plentiful ^1^O_2_. These sufficient active species, especially ^1^O_2_, played a crucial role in photocatalytic NaPCP degradation. NaPCP was effectively degraded mainly through dechlorination, dehydroxylation and benzene ring opening. The present work will provide a novel strategy for the tunable introduction of OVs on semiconductors and insights into the efficient degradation of NaPCP.

## 3. Materials and Methods

### 3.1. Sample Preparation 

All reagents used in this work were of analytical grade and used without further purification. 

BMO-001 was fabricated via hydrothermal synthesis according to our previous work. In detail, 0.242 g of Na_2_MoO_4_·2H_2_O and 0.970 g of Bi(NO_3_)_3_·5H_2_O were dissolved and dispersed in 60 mL of deionized water while stirring continuously. After that, the above suspension was adjusted to pH = 6 using ammonia and then heated at 160 °C for 12 h in the Teflon-lined autoclave. The as-prepared sample was purged with distilled water and ethanol 3 times and finally dried at 60 °C for 12 h.

Bi_2_MoO_6_ with tunable oxygen vacancies was synthesized by calcination treatment. Specifically, a 300 mg BMO-001 sample was placed in a tubular furnace filled with an H_2_/Ar atmosphere, then heated to x °C (x = 200, 250, 300, 350) at 10 °C/min and kept for 2 h, and finally cooled naturally to room temperature. The as-prepared Bi_2_MoO_6_ with oxygen vacancies were named BMO-001-200, BMO-001-250, BMO-001-300, and BMO-001-350.

### 3.2. Sample Characterization 

The X-ray diffraction (XRD) patterns of the as-prepared samples were measured on a Bruker D8 Advance X-ray diffractometer with Cu Kα radiation. A JEOL 6700-F scanning electron microscope (SEM) and FEI TALOS F200 transmission electron microscope (TEM) were utilized to characterize the morphologies and microstructures of these samples. Diffuse reflection spectra (DRS) were acquired using a PerkinElmer Lambda 650s UV/vis spectrometer. The surface electronic states of these photocatalysts were investigated on a Thermo Fisher ESCALAB 250Xi X-ray photoelectron spectrometer (XPS) with all the binding energies referenced to the C 1s peak at 284.6 eV of the surface amorphous carbon. A Bruker MS-5000 electron paramagnetic resonance (EPR) test was performed to detect the OVs and photogenerated reactive oxygen species. A PerkinElmer LS55 was used with steady-state photoluminescence spectra (PL) on the fluorescence spectrometer with an excitation wavelength of 325 nm. An Edinburgh FLS980 was used with time-resolved fluorescence decay spectra (TR-PL) to characterize photoelectron lifetime with a picosecond diode laser excitation wavelength of 375 nm and emission wavelength of 470 nm.

### 3.3. Photocatalytic Activity Test 

The photocatalytic degradation of NaPCP was conducted under the irradiation of the 300 W Xe lamp (PLS-SXE300D, Beijing Perfectlight, λ ≥ 400 nm). In detail, the 50 mg photocatalyst was dispersed in a 50 mL 50 mg/L NaPCP solution in a cylindrical reactor equipped with a cooling system using magnetic stirring. The suspension was first stirred in the dark for 60 min to reach adsorption-desorption equilibrium. During the illumination, about 2 mL aliquot was sampled and filtrated for High-Performance Liquid Chromatography (HPLC) analysis with a UV detector at 227 nm (Agilent 1260). The photocatalytic NaPCP degradation efficiency (η) was calculated using the formula η(%) = (1 − C/C_0_) × 100%, where C and C_0_ were the NaPCP concentrations in the reaction’s aqueous solution.

As for the scavenger system of NaPCP degradation, Triethanolamine (TEOA, 1 mL), isopropanol (IPA, 1 mL), superoxide dismutase (SOD, 1 mg) and natural β-carotene (caro, 1 mg) served as the hole, while •OH, •O_2_^–^ and ^1^O_2_ scavengers were added to investigate the corresponding active species, respectively.

### 3.4. In-Situ DRIFTS Experiments

To further interpret the degradation mechanisms of NaPCP, in-situ DRIFTS experiments were conducted with solid samples on a Thermo Fisher Nicolet iS50FT-IR spectrometer equipped with a designed reaction cell and a liquid nitrogen-cooled HgCdTe (MCT) detector. The mixture of 0.1 g BMO-001-300 and 0.01 g NaPCP was finely ground for 15 min and then deposited on the substrate in the center of the cell. Then, Ar (50 mL/min) was used to purify the cells at 120 °C for 1 h to ensure these impurities on the sample surface dislodged. Subsequently, the mixture of air (30 mL/min) and trace water vapor was introduced into the reactor with the infrared signal in-situ collected by the MCT detector.

## Supplementary Materials

The following supporting information can be downloaded at: https://www.mdpi.com/article/10.3390/ijms232315221/s1. References [40,41,42,43,44,45] are cited in the Supplementary Materials.
