# Supplementary material for: Enhanced Exciton Effect and Singlet Oxygen Generation Triggered by Tunable Oxygen Vacancies on Bi2MoO6 for Efficient Photocatalytic Degradation of Sodium Pentachlorophenol
Source: Int J Mol Sci. 2022 Dec 2;23(23):15221. doi: 10.3390/ijms232315221 (PMC9735721; doi:10.3390/ijms232315221)
Supplement: Supplementary file 1 [file ijms-23-15221-s001.zip › ijms-1988394-supplementary.pdf]

Supplementary material

for

Enhanced exciton effect and singlet oxygen generation triggered by tunable oxygen vacancies on Bi_2MoO_6 for efficient photocatalytic degradation of sodium pentachlorophenol

Xiao Xu[1,2†], Xianglong, Yang[2,3†], Yunlong Tao[2], Wen Zhu[1], Xing Ding[2], Junjiang Zhu[1,] and Hao Chen[2,*]*

1. Hubei Key Laboratory of Biomass Fibers and Eco-dyeing & Finishing, College of Chemistry and Chemical Engineering, Wuhan Textile University, Wuhan 430200, China

2. College of Science, Huazhong Agricultural University, Wuhan 430070, China

3. National Reference Laboratory for Agricultural Testing (Biotoxin), Laboratory of Quality and Safety Risk Assessment for Oilseed Products (Wuhan), Key Laboratory of Detection for Mycotoxins, Quality Inspection and Test Center for Oilseed Products, Ministry of Agriculture and Rural Affairs, Oil Crops Research Institute, Chinese Academy of Agricultural Sciences, Wuhan 430062, China

* To whom correspondence should be addressed. E-mail: jjzhu@wtu.edu.cn; hchenhao@mail.hzau.edu.cn; Phone/Fax: +86-27-59367434

† These authors contributed equally to this work.

4 pages, 5 figures, 2 tables

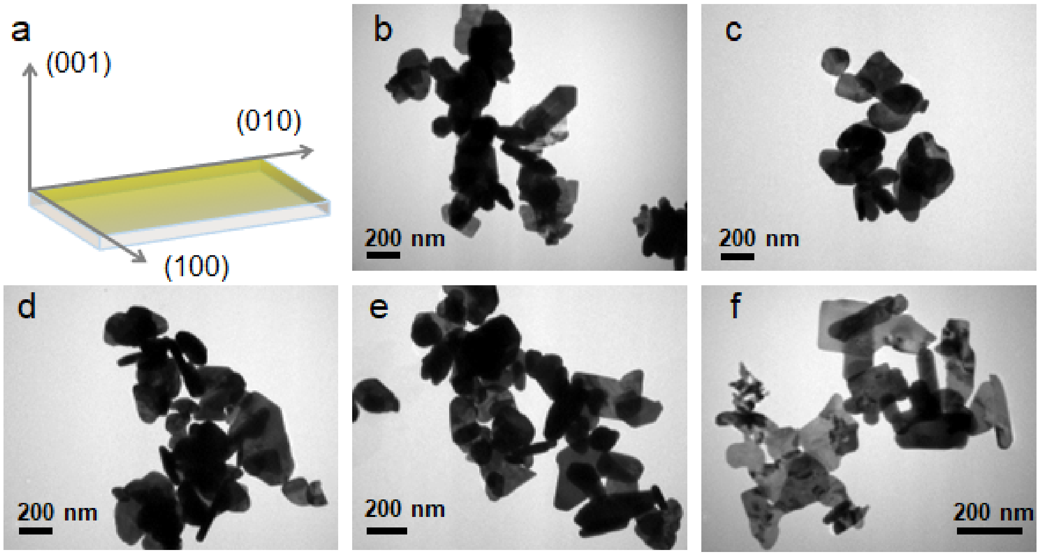

Figure S1. Schematic diagram of Bi_2MoO_6 nanosheets (a) and TEM images of BMO-001 (b), BMO-001-200 (c), BMO-001-250 (d), BMO-001-300 (e) and BMO-001-350 (f), respectively.

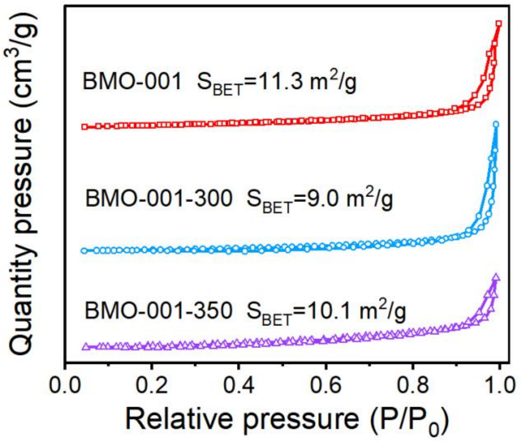

Figure S2. The Brunauer–Emmett–Teller (BET) surface areas of BMO-001, BMO-001-300 and BMO-001-350.

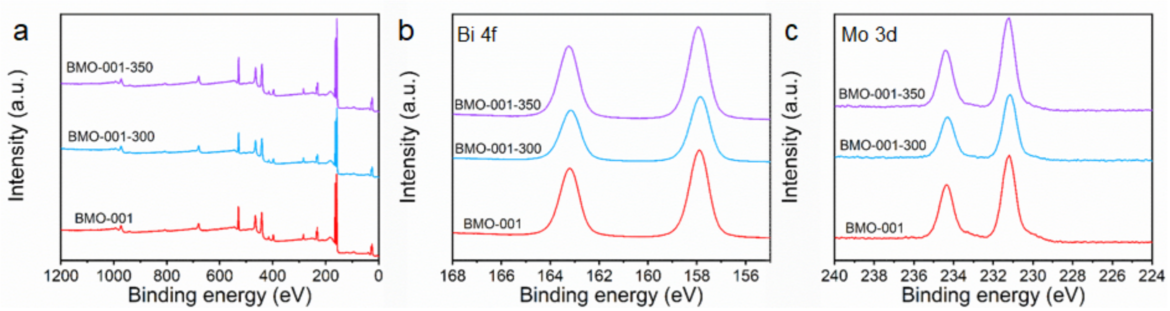

Figure S3. XPS spectra of BMO, BMO-001-300 and Bi-001-350 photocatalysts: (a) survey spectra, (b) Bi 4f and (c) Mo 3d.

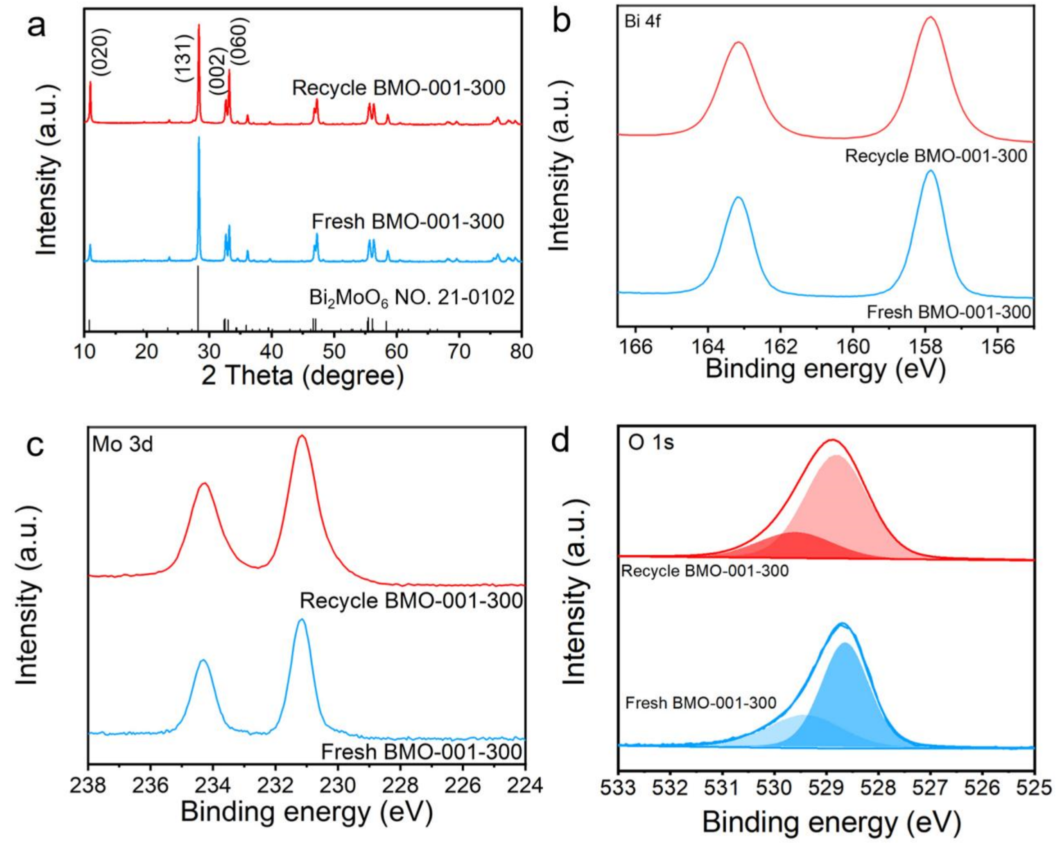

Figure S4. (a) XRD and XPS spectra of (b) Bi 4f, (c) Mo 3d and (d) O 1s over the fresh and recycle BMO-001-300 photocatalysts.

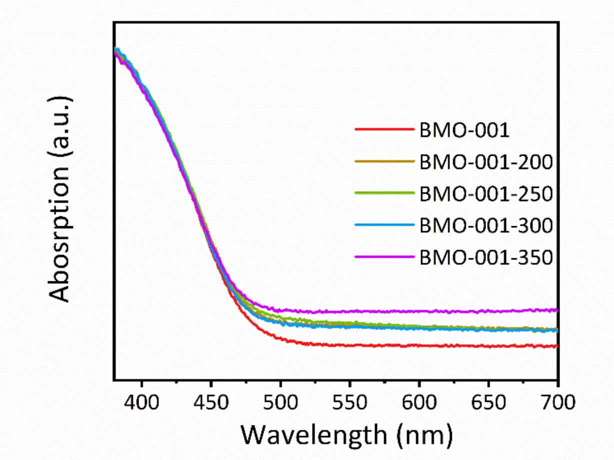

Figure S5. UV-vis diffuse reflectance spectra of as-prepared photocatalysts.

Table S1. Comparison of photocatalytic degradation of chlorophenols pollutants using different photocatalysts.

Photocatalysts	Light resources	Pollutants concentration	Photocatalytic activity	Refs.
$g\text{-}C_3N_4$	300W Xe lamp λ ⩾ 420 nm	10 ppm 4-CP	30.6% within 240 min	[40]
$Au\text{-}C_3N_4$	300W Xe lamp λ ⩾ 420 nm	10 ppm 4-CP	77.2% within 60 min	[41]
$BiOCl\text{-}g\text{-}C_3N_4$	300W Xe lamp λ ⩾ 420 nm	10 ppm 4-CP	95% within 120 min	[42]
$BiO_{2\text{-}X}$	300W Xe lamp λ ⩾ 420 nm	30 ppm 4-CP	100% within 120 min	[43]
TiO_2	500W Xe lamp λ ⩾ 365 nm	20 ppm PCP	85% within 15 min	[44]
$Bi_{2.1}WO_6$	500W Xe lamp λ ⩾ 420 nm	10 ppm NaPCP	91% within 120 min	[45]
Ag/BiOCl	500W Xe lamp λ ⩾ 420 nm	10 ppm NaPCP	80% within 180 min	[46]
Bi/Bi_2MoO_6	300W Xe lamp λ ⩾ 400 nm	10 ppm NaPCP	95% within 120 min	[47]
BMO-001-300	300W Xe lamp λ ⩾ 400 nm	50 ppm NaPCP	80% within 60 min	This work

Note: 4-CP: 4-chlorophenol; PCP: Pentachlorophenol; NaPCP: Sodium pentachlorophenol.

Table S2. The band potential analysis of BMO-001, BMO-001-300 and BMO-001-350.

Sample	Ef vs. Ag/AgCl	Ef vs. NHE	VB-XPS	VB vs. NHE	Eg	CB vs. NHE
BMO	-0.29 V	0.32 V	2.16 eV	2.48 V	2.56 eV	-0.08 V
BMO-001-300	-0.30 V	0.31 V	2.07 eV	2.38 V	2.52 eV	-0.14 V
BMO-001-350	-0.34 V	0.27 V	1.66 eV	1.93 V	2.51 eV	-0.58 V
